# Pancreatic Ductal Adenocarcinoma Arising in Young and Old Patients Displays Similar Molecular Features

**DOI:** 10.3390/cancers13061234

**Published:** 2021-03-11

**Authors:** Jérôme Raffenne, Fernando A. Martin, Rémy Nicolle, Marina Konta, Yuna Blum, Jérôme Torrisani, Francesco Puleo, Jean Baptiste Bachet, Magali Svrcek, Armel Bardier-Dupas, Jean Francois Emile, Peter Demetter, Miroslav Radman, Jean Luc Van Laethem, Pascal Hammel, Vinciane Rebours, Valérie Paradis, Anne Couvelard, Jérôme Cros

**Affiliations:** 1 INSERM U1149, Inflammation Research Center, Beaujon Hospital, 92110 Clichy, France; martin.fernandoariel@gmail.com (F.A.M.); pascal.hammel@aphp.fr (P.H.); vinciane.rebours@aphp.fr (V.R.); valerie.paradis@aphp.fr (V.P.); anne.couvelard@aphp.fr (A.C.); 2 Proteomic Research Group, Mediterranean Institute for Life Science, 21000 Split, Croatia; marina.konta@medils.hr (M.K.); miroslav.radman@gmail.com (M.R.); 3 Programme Cartes d’Identité des Tumeurs (CIT), Ligue Nationale Contre Le Cancer, 75013 Paris, France; remy.nicolle@ligue-cancer.net (R.N.); yuna.blum@gmail.com (Y.B.); 4 Institut National de la Santé et de la Recherche Médicale, INSERM Unit 1037, Centre de Recherches en Cancérologie de Toulouse, CEDEX 1, 31 037 Toulouse, France; jerome.torrisani@inserm.fr; 5 Université Toulouse III-Paul Sabatier, CEDEX 9, 31 062 Toulouse, France; 6 Department of Medical Oncology, Institut Jules Bordet, Université Libre de Bruxelles, 1000 Brussels, Belgium; francesco_puleo@hotmail.com; 7 Laboratory of Experimental Gastroenterology, Université Libre de Bruxelles, 1000 Brussels, Belgium; 8 Department of Gastroenterology, Pitié-Salpetriére Hospital, Sorbonne Universités, UPMC Université, 75013 Paris, France; jean-baptiste.bachet@aphp.fr; 9 Department of Pathology, Saint Antoine Hospital, 75012 Paris, France; magali.svrcek@aphp.fr; 10 Department of Pathology, Pitié-Salpetriére Hospital, 75013 Paris, France; armelle.bardier@aphp.fr; 11 Department of Pathology, Ambroise Paré Hospital, 92100 Boulogne-Billancourt, France; jean-francois.emile@uvsq.fr; 12 Department of Pathology, Erasme Hospital, 1000 Brussels, Belgium; pieter.demetter@bordet.be; 13 Laboratory of Experimental Gastroenterology and Department of Gastroenterology and Digestive Oncology, Hopital Erasme, Université Libre de Bruxelles, 1070 Brussels, Belgium; jl.vanlaethem@erasme.ulb.ac.be; 14 Department of Medical Oncology, Beaujon Hospital—Université de Paris, 92110 Clichy, France; 15 Department of Gastroenterology, Beaujon Hospital—Université de Paris, 92110 Clichy, France; 16 Department of Pathology, Beaujon-Bichat Hospital—Université de Paris, 92110 Clichy, France

**Keywords:** PDAC, young patients, elderly patients, multi-omics

## Abstract

**Simple Summary:**

Pancreatic ducal adenocarcinoma (PDAC) is classically diagnosed in the 7th decade, but approximately 10% of patients are diagnosed under 55 years old (y.o.). Multiple molecular defects such as DNA damages and oxidative stress present in PDAC are associated with ageing. With a multiomics approach, we assessed the molecular features of early-onset tumors (≤55 y.o.) and compared them to classical late-onset tumors (≥70 y.o.). Our results demonstrated that tumors from both groups showed a similar molecular profile. Given that young patients are more often included in clinical trials, this absence of difference is an important finding that supports younger patients as a good molecular surrogate model for the general older population of patients with PDAC.

**Abstract:**

Pancreatic ducal adenocarcinoma is classically diagnosed in the 7th decade, but approximately 10% of patients are diagnosed under 55 years (y.o.). While the genomic and transcriptomic landscapes of late-onset tumors (LOT) have been described, little is known about early-onset tumors (EOT). Ageing is known to impact DNA methylation and proteome integrity through carbonylation-related oxidative damages. We therefore aimed to assess the global molecular features of EOT. We compared 176 EOT (≤55 y.o.) and 316 LOT (≥70 y.o.) from three distinct surgical cohorts at the clinical/genomic/epigenomic/transcriptomic level. Furthermore, we assessed oxidative stress responses and oxidative proteome damages using 2D gel electrophoresis followed by mass spectrometry protein identification. There was no consistent clinical difference between EOT and LOT across the three cohorts. The mutational landscape of key driver genes and the global methylation profile were similar in the two groups. LOT did display age-related features such as enriched DNA repair gene signatures and upregulation of oxidative stress defenses together with increased proteome carbonylation. However, these age-related differences were more preeminent in non-tumor tissues while tumor proteome and proteome damages were fairly comparable. In conclusion, this multi-omics comparison showed that EOT harbor a comparable molecular profile to that of LOT.

## 1. Introduction

Pancreatic ductal adenocarcinoma (PDAC) has one of the worse prognoses, with a very low 5-year survival rate, approximately 7% [1,2]. Predictions put PDAC as the second cause of death by cancer in 2022 [3]. This high mortality rate is due to late-stage diagnosis in most cases and poor chemosensitivity [4]. PDAC is classically diagnosed in the 7th decade (median at 71 years old (y.o.)). However, a subset of patients develops a PDAC in their 4th or 5th decade (about 10% of patients are 55 y.o. or younger at the diagnosis (SEER9, GLOBOCAN 2012 [5]). Data on the clinical behavior and the molecular landscape of early-onset tumors (EOT) are scarce and/or discordant. This is of particular interest as patients included in clinical trials tend to be younger than the “classical” population of PDAC. The good “surrogate” value of early onset patients (EOP) for the global population is therefore critical.

This raises two important questions: (i) what is the clinical and molecular profile of EOT in comparison to LOT? (ii) Are the ageing-related mechanisms implicated in LOT also involved in EOT? He et al. reported no difference in the clinical or pathologic presentation between EOP and late-onset patients (LOP) in a group of resected patients but found that patients younger than age 45 displayed a better overall survival (19 vs. 16 months, *p* = 0.007), a finding similar to that of Duffy et al. [6,7]. In contrast, several studies on consecutive patients (resected and non-resected) suggested that EOP more often present with more advanced stage and shorter overall survival [8,9]. EOP may better tolerate the surgery and the adjuvant treatment but another hypothesis is that EOT have a particular molecular profile.

Very few data on the molecular landscape of these EOT are available, especially on the frequency of the transcriptomic subtypes that have a major prognostic impact [10,11]. All the studies so far were limited to mutational analyses. Mutations of *KRAS* (~90%), *TP53* (~75%), and *CDKN2A* (~35%), and deletion of *SMAD4* (~30%) are the four most common genetic alterations in PDAC [12,13]. Bergmann et al. assessed few mutational hallmarks of PDAC such as *KRAS* and *SMAD4* in seven cases of EOT and reported in this subgroup a lower rate of *KRAS* mutation (3/7) [14]. Recently, Bannon et al. reported a higher prevalence of germline mutations in EOT and a better outcome of these patients possibly due to DNA repair deficiency and increased chemosensitivity [15]. Finally, Ben-Aharon et al. stated that *SMAD4* alterations are more frequent and associated with TGF-β pathway activation in 90 EOT (≤55 y.o.) [16]. These gene mutations are important, as targeted therapies are being developed for specific *KRAS* mutations, and the impact of the *SMAD4* status is still debated but was suggested to be associated with a more local disease and a better prognosis [17]. In addition, Jha et al. reported a different genomic landscape in glioblastoma arising before age 40 with decreased frequency of *TP53* mutation in younger patients, suggesting that some cancer-related events may be age-related [18].

Ageing is associated with a wide range of molecular defects that may play a role in cancer initiation, development, and progression [19]. Ageing impacts telomere length and DNA repair capability that may result in genome instability. In addition, ageing is associated with global methylation that might target tumor suppressor genes and favor tumor initiation [20]. Some of these age-related processes such as telomere shortening are usually “reversed” in tumor cells to favor uncontrolled proliferation. However, because of the different ages of onset, we hypothesize that the mechanisms of cancer initiation and the subsequent tumors might differ in young and old patients, warranting an exploration of their methylation and transcriptomic profiles. Oxidative stress related to hypoxia is a hallmark of PDAC. Similarly, ageing is associated with increased oxidative stress that causes damage to cellular components and leads to their dysfunctions [21]. For instance, protein carbonylation, an irreversible post-translational modification caused by excess oxidative stress, is linked to ageing and may be particularly involved in PDAC [22]. Protein carbonyls induce enzyme loss of activity and hydrophobic aggregates, leading to ageing-associated diseases and promoting cancer [22,23,24,25,26,27]. Early-onset PDAC may therefore develop through alternative mechanisms and may present a different biology, leading to potential new therapeutic targets.

The aim of this study was therefore to compare the clinical and molecular features of PDAC developed in EOP (≤55 y.o.) to PDAC developed in LOP (≥70 y.o.).

## 2. Materials and Methods

### 2.1. Patients and Samples

The hospital ethics committee approved this study (IRB 00003835-2010/01NCIB). The study cohort was composed of 471 consecutive patients with an upfront surgical resection for a PDAC between September 1996 and December 2010 in five academic centers (global population described here [11]). Patients were selected in the early-onset cohort if they were younger than 55 y.o. (≤55) at the time of surgery (*n* = 106) and in the late-onset cohort of they were older than 70 y.o. (≥70) at the time of surgery (*n* = 149). Very early-onset patients (VEOP) were defined as being diagnosed before the age of 45 years (*n* = 18). Classical clinical and pathological data were collected.

For the proteomic analysis, samples were provided by the Beaujon Hospital Pathology cryo-library (Biobanque NFS9600). All the tissue samples were reviewed by a pathologist. All the non-tumor samples with fibrosis and/or inflammation were excluded. All the tumor samples with a cellularity below 40% were also excluded. Selected samples were dispatched in two groups: patients ≤ 55 y.o. (10 patients) and ≥70 y.o. (14 patients). When possible, non-tumor and tumor tissues from the same patient were analyzed.

Two additional public cohorts were also analyzed with the same selection criteria: (i) 87 primary tumors (30 EOP and 57 LOP were selected from the curated TCGA_PAAD cohort (TCGA Research Network: http://cancergenome.nih.gov/ (accessed on 22 January 2021)) and 150 primary tumors (40 EOP and 110 LOP) from the curated ICGC_PAN_AU cohort [13,28]. In all these cohorts, neuroendocrine tumors or carcinomas, mixed tumors, and acinar cell carcinomas were excluded. In addition to clinical data, methylation and transcriptomic data were retrieved and analyzed

### 2.2. DNA-Based Analyses

In our cohort, the tumor DNA was available for 142/255 patients (≤55 y.o. *n* = 53 and >70 y.o. *n* = 89). DNA was extracted from the formalin-fixed paraffin-embedded tissue blocks that were also used to extract the RNA used in the microarray study. The QiaAMP DNA FFPE tissue kit (Qiagen, Courtaboeuf, France) was used according to the manufacturer’s instructions. Next-generation sequencing libraries were prepared using Ion AmpliSeqTM Cancer HotSpot Panel v2 (Life Technologies, Courtaboeuf, France) following the manufacturer’s recommendations. Torrent Variant Caller Plugin was used to determine target variants (substitution, insertion, or deletion). A sequence variation was considered as a mutation (i) if the coverage was higher than 200, (ii) the variation was not reported in the 1000 genomes database, and (iii) if the variation was a missense or a nonsense variation.

Genomic and methylation data were retrieved for the curated TCGA_PAAD (*n* = 150, RNAseq) and the ICGC_PAN_AU series (*n* = 264, microarrays).

### 2.3. RNA-Based Analyses

In our cohort, the tumor transcriptomic profile was available for 156/255 patients (≤55 y.o. *n* = 62/106 and ≥70 y.o. *n* = 105/149). RNA profiles were obtained from Affymetrix microarrays as previously described [11]. Data were analyzed using the gene pattern software suite. Gene Set Enrichment analysis used the broad institute software GSEA and Molecular Signature Database (MSigDB). Similar analyses were performed on the transcriptomic data from the TCGA_PAAD cohort (≤55 y.o. *n* = 30 and ≥70 y.o. *n* = 57) and the ICGC_PAN-AU cohort (≤55 y.o. *n* = 40 and ≥70 y.o. *n* = 110). As datasets were not compared directly one with another, the normalized gene expression levels provided by the repositories were used with no further normalization.

### 2.4. Proteomic Analyses

#### 2.4.1. One-Dimension Experiments

Fifty mg of pancreatic tissue was ground in a mortar and dissolved in UTC Buffer (Urea 8 M, Thio-urea 2M, CHAPS 4%, (Sigma Aldrich, St. Quentin Fallavier, France)), pH 7.4, overnight at 10 °C with vigorous shaking. Samples were centrifuged at 21,000× *g* for 2 h, and the supernatant was recovered. The amount of recovered proteins was quantified by the Bradford method. Total protein carbonylation was evaluated between each sample as the difference in gel in one dimension (1D-OxyDIGE). 1D-OxyDIGE experiments were carried out as follows: 50 µg of each protein extract in lysis UTC buffer, pH 4, was incubated 60 min with 1 mM of cyanine-labelled hydrazine (Cy-Hz) (92165, Insight Biotechnology Ltd., Wembley, UK) in an agitated thermomixer at 4 °C protected from light. To stop the reaction, 25 µL of UTC buffer at pH 8 was added. To eliminate excess Cy-Hz and to concentrate the proteins, the reaction volumes were transferred to a centrifugal filter device with a lower cut-off of 10 kDa (Amicon^®^ Ultra-0.5, Merck Millipore, Molsheim, France) according to the manufacturer’s instructions. The total recovery volume was denaturized with 1/10e β-mercaptoethanol and 1/5 loading buffer, 95 °C 10 min, before separation in SDS-PAGE 4–12%-stain free (Mini-PROTEAN^®^ TGX Stain-Free™, Biorad, Marnes-La-Coquette, France). Protein-derived Cy-Hz was visualized in a Typhoon™ FLA 7000 (GE Healthcare) before “stain free” acquisition using a ChemiDoc touch (Biorad) apparatus. The level of total protein carbonylation was normalized to total protein acquisition (“Stain free” technology, Biorad^®^) in gel.

#### 2.4.2. Two-Dimension Experiments

To determine deregulated and carbonylated proteins in tissue samples, 2D-OxyDIGE experiments were carried out. For each protein extract, removal of contaminant product was performed using the 2D Clean Up kit (GE Healthcare). One hundred µg of proteins was labelled with a 0.12 mM solution of Cy-Hz. Excess unreacted dye was removed by TCA precipitation. The pellets containing the carbonyl labelled proteins were re-suspended at a concentration of 5 µg/µL. Fifty µg of carbonyl-labelled protein per sample was minimally labelled using G-Dye 200. An internal standard (IS) was prepared with equivalent amounts of each pooled sample and labeled with G-Dye-100. Fifty µg of each sample and 50 µg of the IS were mixed together and separated by isoelectrofocusing in a pH 3–11 NL, 24 cm Immobiline DryStrip (GE Healthcare Life Sciences, Illkirch, France). Strips were equilibrated in a DTT solution followed by an iodo-acetamide solution. Second-dimension experiments were performed using an 8–18% acrylamide-bis acrylamide gel. Once finished, gels were scanned in an Ettan DIGE Imager (GE Healthcare, Little, Chalfont, UK) and images analyzed using 2D SamesSpot Analysis software (Totallab Ltd., Newcastle-Upon-Tyne, England). Images were aligned to the Internal Standard Images (Cy2NHS channel). Significant differentially expressed spots (Cy3 Channel 1·5-fold change, *p* ≤ 0.05) and differentially carbonylated spots (Cy5 channel 1·3-fold change, *p* ≤ 0.05) were detected between tumor vs. non tumor groups and selected for analysis. Each excised spot of protein from the 2D-OxiDIGE experiment was run in an LTQ-ORBITRAP-Velos for MS/MS analysis. Pathway analyses of the differentially expressed proteins were carried out using the STRING and ENRICHR databases [29,30].

#### 2.4.3. Western Blot Analysis

Proteins were extracted from tumor and non-tumor pancreatic tissues in a lysis buffer (Tris-HCl 4 mM, EDTA 100 mM, EGTA 100 mM, Triton X-100^®^ 1%, Protease and Phosphatase inhibitor cocktail 0.5% each, pH 7). Cellular debris was removed by centrifugation at 20,000× *g* for 10 min. The protein concentration was determined by the method of Bradford by using bovine serum albumin as the standard. Ten micrograms of each extract was separated by 4-12-SDS-PAGE, transferred onto a PVDF membrane, saturated with 5% BSA in Tris-Buffered Saline buffer with Tween-20^®^ 0.5%, pH 7.5 (TBST-BSA) overnight, and blotted with the appropriate primary antibody (Supplementary Table S1). After three washes with phosphate buffer saline (PBS, Gibco, Life Technologies, Courtaboeuf, France), the membranes were incubated with the appropriate secondary antibody (Table S1) for 1 h at room temperature. Image acquisitions were carried out with a Chemidoc apparatus (Biorad) after incubating the membrane in an electro-chemiluminescence buffer (Clarity, Biorad).

### 2.5. Statistical Analyses

We used non-parametric tests (Chi2 and Fisher’s exact tests) to compare independent groups for categorical data and the Kruskall–Wallis test for continuous data. Correlation between gene expressions was assessed using the Pearson correlation coefficient. The outcome variables were progression-free survival (PFS) and overall survival (OS). PFS was defined as the time interval between the day of surgical resection and the date of local or regional relapse, or occurrence of distant metastases, or appearance of second PDAC, whichever occurred first. Survival data were censored at the last follow-up. OS was calculated as the time interval from the day of surgical resection to death (all causes) or until the date of the last follow-up, at which point data were censored. Survival curves were estimated using the Kaplan–Meier method, and differences among groups were analyzed using the log-rank test with Med-Calc^®^. Statistical analyses were carried out using GraphPad Prism 6^®^. For age-specific DNA methylation, CpG methylation was tested by comparing the beta-values in the two age groups using Student’s t-test after removing CpG targeting probes associated with polymorphisms. Only CpG in autosomes was tested. No CpG was found to be associated to the tested age groups at a 5% FDR. Methylation heatmaps were drawn using the 1000 most variable (high standard deviation) CpGs in each series.

## 3. Results

### 3.1. Clinico-Pathological Characteristics of Early-Onset Patients

Two hundred and fifty-five patients that matched our inclusion criteria were selected from the multi-centric cohort (106 younger than 55 and 149 older than 70 y.o.). The comparison of the clinical features is reported in Table 1. Except for a higher rate of vascular invasion in EOT (53.7% vs. 38.9% *p* = 0.024), there was no difference in sex, pTNM/stage or tumor differentiation. Vascular invasion was associated with only a shorter overall survival in EOP (Table 2). In LOP, the stage together with lymph node status and tumor differentiation was associated with a shorter progression-free survival in univariate analysis (*p* = 0.023; 0.0007 and 0.015, respectively). In multivariate analysis, tumor differentiation and the lymph node status in LOP retained a statistically significant value (*p* = 0.008 and 0.002, respectively). Only the lymph node status was associated with a poor prognosis in the LOP cohort (HR = 2.043; CI95% 1.4–4.16, *p* = 0.002). In the very-early onset patients (VEOP) (<45 y.o., *n* = 18), tumor size was an independent poor prognosis factor (Table S2). There was no difference in progression-free or overall survival between EOP and LOP (Figure S1a,b). VEOP tended to have a longer overall survival (38 vs. 29 months) but this was not statistically different (data not shown). These observations were confirmed in part in the pooled TCGA_PAAD and ICGC_PAN_AU cohorts (Tables S3 and S4 and Figure S1c,d).

### 3.2. Genomic and Methylomic Landscape of PDAC in Early-Onset Patients

**Figure 1:**
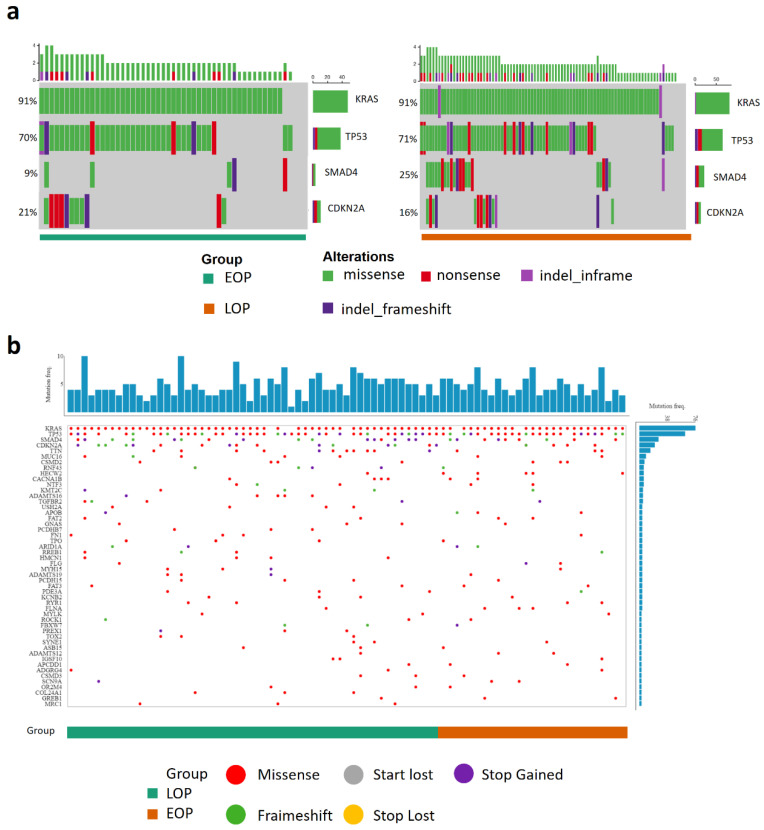
Genomic landscape of pancreatic cancer patients. (**a**) Oncogrid of the top four altered genes (*KRAS*, *TP53*, *SAMD4*, and *CDKN2A*) in the Multi-centric cohort, 53 tumors from early-onset patients (EOP), 89 tumors from late onset patients (LOP); (**b**) Oncogrid from the TCGA data portal web application of the TCGA_PAAD project composed of 87 patients, 30 tumors from EOP (orange) and 57 tumors from LOP (green). Genes are ordered by decreasing alteration frequencies in each tumor. Each type of gene mutation is represented as a colored dot, red: Missense mutation, grey: loss of the start codon, purple: gain of the stop codon, green: coding sequence frameshift, yellow: loss of the stop codon.

We compared the mutational landscape in 53 EOP and 89 LOP of our cohort (detailed sequencing results are shown in Table S5). There were no differences in the main driver gene mutation frequencies (*KRAS*, *TP53*, and *CDKN2A*) between EOP and LOP. LOP presented more frequent *SMAD4* mutations (22/89 (24.7%) vs. 5/53 (9.4%), *p* = 0.043) (Figure 1a and Table 3). This result was not confirmed in the ICGC_PAN_AU cohort (no difference in the mutation frequency in *KRAS*, *TP53*, *CDKN2A*, and *SMAD4*). Copy number variation (CNV) data were available for the TCGA_PAAD cohort in addition to the mutational status. However, there was no difference in the genomic landscape between EOP and LOP or VEOP and LOP (Figure 1b and Figure S2).

DNA methylation is one of the key mechanisms that control gene expression and was reported to be modified through ageing [31,32]. We investigated whether ageing influenced the methylation profile of PDAC in the TCGA_PAAD and ICGC_PAN AU cohorts. There was no difference in the CpG methylation profile of EOT and LOT in the TCGA_PAAD (Figure S3a) and the ICGC_PAN AU cohorts (Figure S3b). We also investigated the patterns of CpG methylation but found no difference (Figure S3c).

### 3.3. Transcriptomic Landscape in Early-Onset Patients

**Figure 2:**
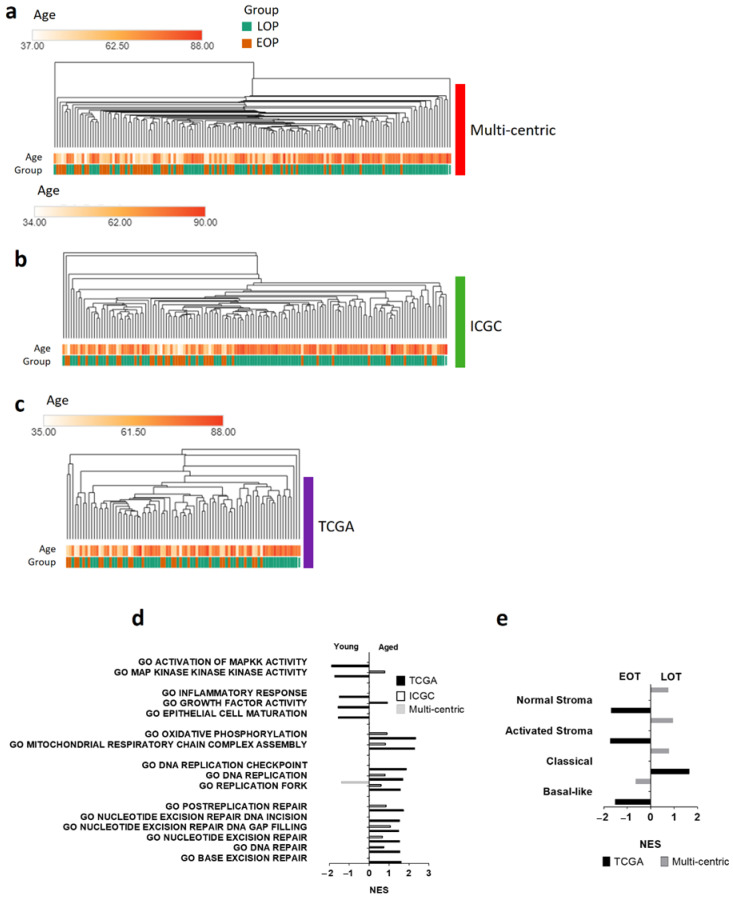
Transcriptomic landscape of early-onset patients (EOP) and late-onset patients (LOP). Hierarchical clustering of EOP (orange) and LOP (green) from (**a**) Multi-centric cohort; (**b**) ICGC_PAN_AU cohort; (**c**) TCGA_PAAD cohort. (**d**,**e**) Gene set analysis (GSEA) of LOP vs. EOP from each dataset. Normalized Enrichment Score (NES) with a false discovery rate less than 0.25, and a *p* value less than 0.05. (**d**) GSEA of gene ontology (GO); (**e**) GSEA of Moffitt et al. PDAC subtype signature [10].

To further study early-onset PDAC, we compared the transcriptomic profile of EOT and LOT patients. Unsupervised analysis showed no age-related clustering, and we found similar results in the TCGA_PAAD and the ICGC_PAN_AU cohorts (Figure 2a–c). Similar results were obtained with the VEOP (Figure S4). To assess the pathways activated in each group, we performed gene set enrichment analyses of the three datasets. None of the pathways were consistently enriched in all three datasets (Figure 2d). However, we found signatures related to DNA repair mechanisms (NES > 1.3, *p* < 0.05) and oxidative phosphorylation enriched in LOT in both the TCGA_PAAD and the ICGC_PAN_AU cohorts. EOT presented, but inconsistently across the three datasets, enriched signatures for hypoxic response, hedgehog signaling, inflammatory response (TNF-α signaling), up-regulation of *KRAS* signaling, and epithelial to mesenchymal transition. These signatures, classically associated with aggressive PDAC, were in concordance with the enrichment of the aggressive “basal-like” PDAC subtype in EOT in contrast to the enrichment of the “classical subtype” in LOT (Figure 2e). There was no difference in the enrichment of the stromal subtypes described by Moffitt et al. [10] in the EOT or LOT groups (Figure 2e).

### 3.4. Oxidative Stress Damages and Defenses in Early-Onset Patients

**Figure 3:**
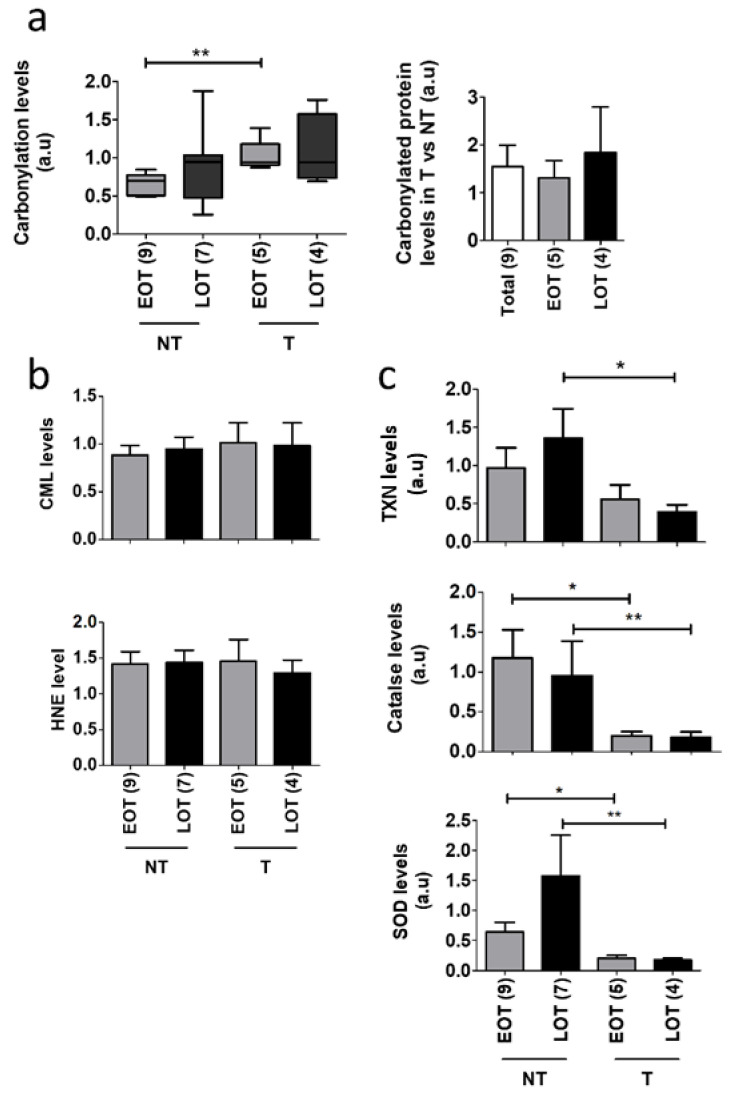
Oxidative stress damages in EOT vs. LOT. (**a**) Left panel: In-gel analysis of total protein carbonylation levels of non-tumor (NT) and tumor tissues (T) from early-onset patients (EOP) and late-onset patients (LOP) normalized by total protein input. Right panel: carbonylated protein rates of T compared to NT in EOP and LOP. Representative in-gel protein deposits are displayed in Figure S5a; (**b**) N(6)-Carboxyl-methyl-lysine (CML) and 4-Hydroxynonenal (HNE) levels quantified by Western blot and normalized by total protein expression (“stain-free” Biorad^®^ technology). Representative stain-free membranes, HNE, and CML Western blots are displayed in Figure S5b,c, (**c**) Thioredoxin (TXN), catalase, and superoxide dismutase (SOD) antioxidant protein expression normalized to β-Actin expression was analyzed by Western blot from EOP and LOP. Representative blots are displayed in Figure S5d, *t* test: * *p* < 0.05; ** *p* < 0.01.

Oxidative stress is a hallmark of ageing and is involved in pancreatic cancer progression. We analyzed the global ROS-related alterations of the proteome, i.e., protein carbonylation. We compared 10 EOT and 14 LOT with available non-tumor adjacent tissue (no clinically significant differences between the two groups (Table S6). In PDAC, global carbonylation was slightly increased, albeit not statistically different, in tumor and non-tumor tissues of LOP (Figure 3a and Figure S5a). Tumor tissues compared to non-tumor tissues showed an increased level of global carbonylation but this was only statistically significant in EOT (1.31 fold, *p* = 0.0035 in EOT, 1.84 fold, *p* = 0.52 in LOT). We also assessed two other markers of oxidative stress, the advanced glycation end product N(6)-Carboxyl-methyl-lysine (CML) and 4-Hydroxynonenal (HNE) that is produced through lipid oxidation and found no differences between EOT and LOT (Figure 3b and Figure S5b). To better understand the increased ROS-mediated damages in tumor tissues, we assessed the protein expression of three major antioxidant proteins, super oxide dismutase (SOD), catalase, and thioredoxin (TXN). In non-tumor tissues, the level of SOD and TRX was increased in LOT but this was not observed in tumor tissues (Figure 3b and Figure S5d). In addition, the level of these three antioxidant proteins was decreased in tumor compared to non-tumor tissues.

### 3.5. Protein Oxidative Damages in Early-Onset Patients

**Figure 4:**
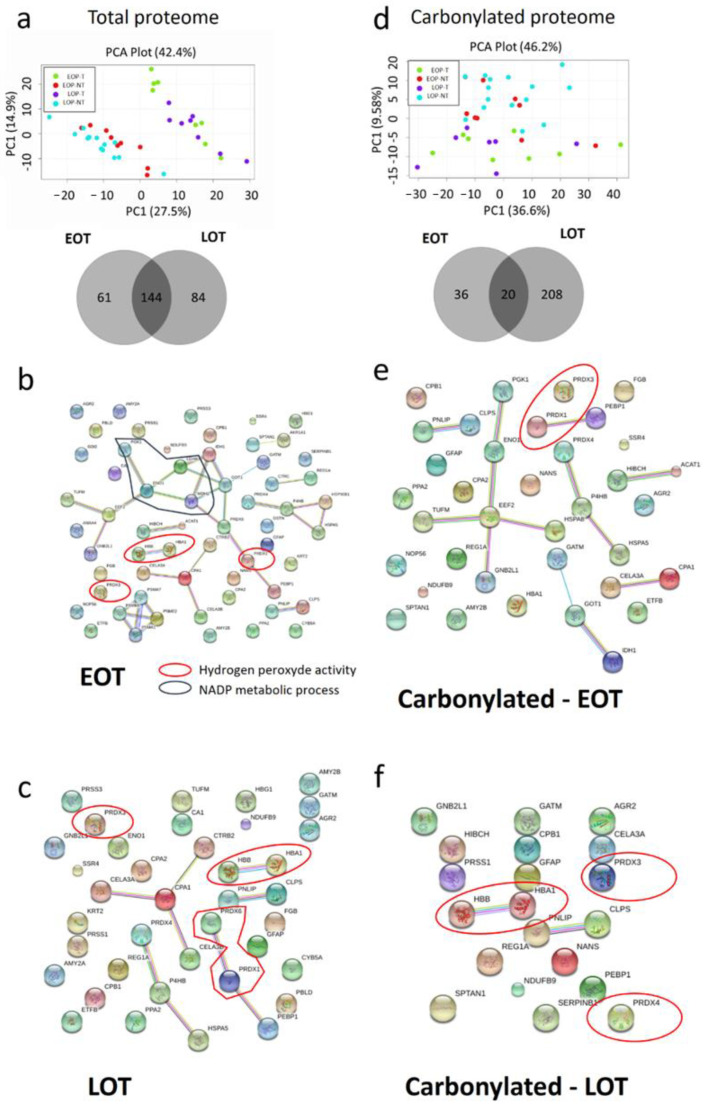
Specific protein damages in tumor tissues (T) compared to non-tumor tissues (NT) in early-onset patients (EOP) and late-onset patients (LOP). (**a**) Principal component analysis (PCA) of total protein expression from each spot analyzed by 2D-DIGE. Lower panel: Venn diagram of specific spots from EOP and LOP, (**b**) PCA of carbonylated protein expression from each spot analyzed by 2D-DIGE. Lower panel: Venn diagram of specific spots from EOP and LOP. (**c**–**f**)—String software analysis of significant differential proteins (**c**) Down regulated in EOP; (**d**) Down regulated in LOP; (**e**) More carbonylated in EOP; (**f**) More carbonylated in LOP.

Since our one-dimension protein damage analysis was only quantitative, we performed a two-dimension gel electrophoresis analysis to identify by mass spectrometry the proteins and their carbonylation status in EOT and LOT. In total, 640 peptides spots were analyzed. Tumor and non-tumor proteomes were qualitatively distinct with a clean separation on the principal component analysis, but age did not separate tumor or non-tumor tissues (Figure 4a). A similar observation was made for the carbonylated proteome with a less clear separation of tumor and non-tumor samples (Figure 4c). Some peptidic spots were specifically dysregulated in EOT or LOT groups (61 and 84, respectively) (Figure 4a). Similarly, age-specific carbonylated peptidic spots were more abundant in LOT (*n* = 208) than in EOT (*n* = 36) (Figure 4d). We selected the 120 most significantly deregulated peptidic spots with at least ± 1·2-fold, *t* test *p* < 0.05, and identified the corresponding proteins (Table S7). Only 18 proteins were significantly differentially expressed in the tumor compartment of EOP and LOP (increased in EOT), with none in the non-tumor compartment. EnrichR pathway analysis showed that the differentially expressed proteins were aggregated in the TriCarboxylic Acid cycle (TCA) (Adj. *p*-value. < 10^−4^). We also compared the pathway activated in EOT and LOT compared to their respective non-tumor tissues. This revealed a similar pattern in both populations with a decrease in hydrogen peroxide activity (downregulation of peroxiredoxin (PRDX) 1, 3, 4 and 6, and TXN) compared to non-tumor tissues (Figure 4b,c). In addition, these ROS-handling proteins were also more carbonylated in tumor tissues in both EOT and LOT (Figure 4e,f).

## 4. Discussion

Oxidative stress is a hallmark of ageing. Reactive species, if improperly handled, can damage most cellular components, especially DNA, leading to mutation and chromosomal instability or proteins leading to inactivation through carbonylation [21,33,34] In light of this, in some aspect, PDAC with its high level of oxidative stress could be considered an age-related disease. This is in line with the classical late onset of the disease, in the 7th decade. Early-onset PDAC is therefore peculiar and is most often diagnosed with no particular familial history, suggesting that germline predisposing mutations cannot explain all cases. In addition, age was shown to impact the carcinogenesis mechanisms in some cancer types such as glioblastoma [18]. To understand the mechanisms that drive EOT, we performed a multi-omics comparison of EOT and LOT and showed no difference in the clinical outcome, the mutational landscape, and the methylation profile. Repartition of the transcriptomic subtypes was similar in the two groups. LOT display mild age-related features such as enriched DNA repair gene signatures and upregulation of oxidative stress defenses together with increased proteome carbonylation. However, these age-related differences were more preeminent in non-tumor tissues, and tumor proteome damages were fairly comparable between the groups.

Whether EOT have particular clinical features and outcome is still unclear. Eguchi et al. and Tingstedt et al. reported on unselected cohorts (i.e., resectable and advanced PDAC); overall, EOT are diagnosed at a more advanced stage with a poorer prognosis [8]. In the subgroup of resected patients, they found a comparable prognosis while others reported a better survival of early-onset patients with stage I-II disease [6,7]. These discrepancies could be explained by the differences in disease stage in the studied populations and possibly by the slight increased frequency of germline mutations in DNA repair genes in EOT that are known to sensitize tumors to platinum-based therapies. In our large multi-centric cohort of consecutive resected PDAC, we found few pathological differences between EOT and LOT that were not consistently found in the two additional cohorts. In all three cohorts, patients with EOT and LOT had similar progression-free and overall survival and these results were similar if we decreased the lower age limit to 45 years, although the size of the cohort was small. Most patients received gemcitabine-based regimens and these findings will need to be re-evaluated on more recent cohorts treated by platinum-based therapies that may be better tolerated by younger patients.

There were almost no molecular data on EOT until the recent report by Ben-Aharon et al. [16]. They reported for an unselected cohort a similar genomic profile of EOT and LOT with fewer *KRAS* but more *SMAD4* alterations in EOT. We did not find similar results in our cohort oor in TCGA_PAAD or the ICGC_PAN_AU. One possible explanation is that our cohorts are constituted only of resected samples. We could not dissociate local from advanced patients in the report from Ben-Aharon et al. to confirm this hypothesis. This is particularly important for *SMAD4*, as its loss was related to distant rather than local spread [35,36]. Another potential explanation is that our sequencing panel only identified mutations and not copy number variation (CNV), an important feature for tumor suppressor genes such as *SMAD4;* still, in the TCGA_PAAD cohort, for which the CNV data were available, we could not confirm age-related differences in *SMAD4* alterations. This discrepancy might also come from the use of the uncurated TCGA_PAAD cohort by Ben-Aharon et al. that includes non-PDAC samples such as neuroendocrine lesions. Unfortunately, samples in the repositories are most often mislabeled. The final list of PDAC samples (*n* = 150 out of the 176 to 185 depending on the repository) was reported in the seminal TCGA_PAAD publication [37]. The use of the uncurated cohort was shown to potentially induce bias in the results, as non-PDAC samples have a different biology and prognosis [38,39].

While ageing was shown to alter the global methylation pattern of tissues, global DNA methylation analysis showed no age-specific difference in PDAC [32,40]. Unsupervised transcriptomic analyses showed that age by itself does not sort patients into separate groups. However, gene set enrichment analyses showed an increase in DNA repair signatures in LOT. This could be explained by the fact that ageing is associated with the loss of DNA repair fidelity and with increased oxidative stress-related DNA damages [41,42]. In contrast to what was reported by Ben-Aharon et al., we did not observe an enrichment of the TGF-β signature in EOT datasets. This could be explained by the different SMAD4 mutation distributions in our groups compared to theirs. Our lower rate of alteration could explain the absence TGF-β SMAD4-independent pathway activation in our study. One limitation is that we used microarrays rather than RNA equencing. While microarrays do have a lower dynamic range that is unfavorable to low expressed genes, it is well adapted to degraded formalin-fixed paraffin-embedded samples. Our validation cohorts were composed of RNAseq (TCGA_PAAD) and microarrays (ICGC_PAN_AU) and we did not observed a technology bias in our comparison.

Ageing is associated with an increased ROS production that, besides DNA, targets lipids and proteins. While we did not detect differences in lipid oxidation, we observed a mild increase in protein carbonylation in LOT albeit not statistically significant. The carbonylation level heterogeneity in non-tumor and tumor tissues was greater in LOT, suggesting an important inter-individual variation in ROS-handling capacities. As reported in other tumor types, the carbonylation level was greater in tumor compared to non-tumor tissues [25,27,43]. In PDAC, this could be explained by the important tumor hypoxia generating a high level of ROS. In addition, tumor tissues displayed an age-independent loss of several ROS handling proteins. Similarly to the transcriptomic analysis, the unsupervised analysis of the global proteome showed no age-related groups. While we found more carbonylated spots in LOT than EOT, only few age-specific proteins could be identified by mass spectrometry, and pathway enrichment revealed no differences between EOT and LOT when compared to their respective non-tumor tissues. Taken together, these results suggest that protein damages are increased in tumors regardless of the patient age and that both EOT and LOT deal similarly with excess ROS. A limitation of this study is that two-dimension gel coupled to LC-MS is a powerful approach for protein carbonylation study but it may lead to lower differential protein identification compared to label-free techniques.

## 5. Conclusions

In conclusion, we highlighted in this study that the molecular features of PDAC patients are independent of ageing. Given that young patients are more often included in clinical trials, the absence of a difference is an important finding as it shows that young patients are a relevant study population for the general population of older patients with PDAC.

## Figures and Tables

**Table 1:** Clinical features of the multi-centric cohort of this study.

Cohort Features *n* = 255				
Age Group		EOP < 55 y.o. (106)	LOP > 70 y.o. (149)	*p*
Sex (*n* = Men (%)) *n* = 255		46 (43.39%)	75 (50.33%)	0.389 ⁱ
Age (median (min-max)) *n* = 255		51 (34–55)	74 (70–88)	<0.0001 ⁱ
T *n* = 255				0.841 *
	T1	4	4	
	T2	16	25	
	T3	86	120	
N *n* = 255	N0/N1	32/74	35/141	0.292 *
Tumor differentiation *n* = 255				0.197 *
	Well	35	68	
	Moderate	48	50	
	Poorly	20	27	
	Unknown	3	4	
Tumor size *n* = 238 (mm mean—CI95)		31.9 (27.8–35)	33.9 (31.0–36.8)	0.239 ⁱ
Vascular invasion *n* = 204 (*n* (%))		57 (53.7)	58 (38.9)	0.024 ⁱ
Perinervous invasion *n* = 237 (*n* (%))		81 (76.6)	104 (69.8)	0.346 ⁱ

*: Chi square test. ^i^: Student’s *t* Test.

**Table 2:** Uni- and Multivariate analysis of Overall survival (OS) and Progression-free (PFS) survival of EOP and LOP from our multi-centric cohort.

Cohort features *n* = 255	Univariate PFS EOP <55 y.o. (106)			Univariate PFS LOP >70 y.o. (149)			Multivariate PFS LOP >70 y.o. (149)			Univariate OS EOP <55 y.o. (106)			Univariate OS LOP > 70 y.o. (149)		
Age Group	HR	95% Ci	*p* *	HR	95% Ci	*p* *	HR	95%Ci	*p* *	HR	95%Ci	*p* *	HR	95%Ci	*p* *
**Sex (Men)**	0.79	0.49–1.27	0.335	0.841	0.56–1.26	0.405				0.956	0.58–1.57	0.86	0.786	0.52-.13	0.187
**Age**	1.023	0.97–1.07	0.396	0.944	0.88–1.00	0.084				1.013	0.96–1.06	0.638	0.985	0.93–1.04	0.611
**T**						0.023						0.954			
**T1**	0.883	0.27–2.81	0.834	0.2	0.02–1.42	0.11	0.326	0.04–2.39	0.273	1.131	0.35–3.62	0.837	0.266	0.03–1.89	0.189
**T2**	0.656	0.32–1.32	0.24	0.583	0.32–1.04	0.072	0.706	0.38–1.28	0.255	0.931	0.47–1.83	0.837	0.716	0.42–1.22	0.223
**T3**	1	1	1	1	1	1	1	1	1	1	1	-	1	1	1
**N0/N1**	1.639	0.96–2.79	0.071	2.697	1.52–4.75	0.0007	2.53	1.11–4.54	0.002	0.571	0.32–1.02	0.06	0.415	0.24–0.71	0.002
**Tumor differentiation *n* = 255**						0.015									
**Well**	0.875	0.50–1.50	0.634	1	1	1	1	1	1	0.942	0.52–1.68	0.841	1	1	1
**Moderate**	1	1	1	1.787	1.13–2.82	0.013	1.738	1.09–2.76	0.02	1	1	1	1.18	0.76–1.83	0.459
**Poorly**	1.215	0.65–2.26	0.542	1.966	1.14–3.38	0.015	2.14	1.23–3.71	0.006	1.343	0.696–2.59	0.382	1.65	0.96–2.83	0.068
**Unknown**	0.841	0.20–3.49	0.813	0.451	0.06–3.25	0.432	0.893	0.11–6.77	0.913	1.525	0.46–5.00	0.488	0.728	0.17–2.99	0.662
**Tumor size**	1.005	0.99–1.01	0.436	1.008	0.99–1.02	0.147				1.003	0.98–1.02	0.747	1.008	0.99–1.01	0.122
**Vascular invasion**	1.43	0.99–2.0	0.054	1.35-	0.88–1.45	0.322				1.508	1.03–2.19	0.032	1.064	0.83–1.36	0.619
**Perinervous invasion**	1.246	0.72–2.13	0.425	1.42	0.98–2.07	0.061				0.999	0.57–1.75	0.999	1.295	0.90–1.85	0.158

*: Cox proportional hazard regression.

**Table 3:** Genomic landscape of the multi-centric ICGC and TCGA from EOP and LOP.

Mutations	Multi-Centric			ICGC			TCGA		
	EOP (53)	LOP (89)	p *	EOP (39)	LOP (110)	p *	EOP (27)	LOP (55)	p *
*KRAS n* = (%)	48 (90.5)	81 (91)	0.832	29 (74.3)	87 (79.1)	0.653	25 (92.6)	50 (90.9)	0.704
*TP53 n* = (%)	37 (69.8)	63 (70.8)	0.946	19 (48.7)	70 (63.6)	0.129	23 (85.2)	39 (70.9)	0.183
*CDKN2A n* = (%)	11 (20.7)	14 (15.7)	0.594	4 (10.2)	15 (13.6)	0.781	16 (59.2)	33 (60)	1.00
*SMAD4 n* = (%)	5 (9.4)	22 (24.7)	0.043	7 (17.9)	27 (24.5)	0.507	12 (44.4)	22 (40)	0.812

*: Chi square test.

## Data Availability

Publicly available datasets were analyzed in this study. The TCGA Pancreatic cancer project (PAAD) can be found here: https://portal.gdc.cancer.gov/ (accessed on 22 January 2021). The ICGG Pancreatic cancer project (PACA_AU) can be found here: https://dcc.icgc.org/ (accessed on 22 January 2021). Data from proteomics analysis are available upon reasonable request.

## Supplementary Materials

The following are available online at https://www.mdpi.com/2072-6694/13/6/1234/s1, Figure S1: Overall and Progression-Free Survival of EOP and LOP from (a–b) the multi-centric cohort and (c–d) the TCGA_PAAD/ICGC_PAN_AU combined cohorts, Figure S2: Genomic landscape of pancreatic cancer patients. TCGA_PAAD; 66 patients, 9 VEOT and 57 LOT. Each type of gene mutation is represented as a colored dot. Online analysis TCGA data portal, Figure S3: Methylome profiles of patients from (a) the TCGA_PAAD full cohort (*n* = 150) and (b) the ICGC_PAN_AU full cohort (*n* = 268). (c,d) Count of CpG dimers in the function of methylation rates in EOT (c) and LOT (d), reported as b-value in the TCGA_PAAD cohort, Figure S4: Transcriptomic landscape of VEOP (<45 y.o.) vs. LOP (>70 y.o.). Hierarchical clustering of VEOP and LOP from (a) Multi-centric cohort; (b) ICGC_PAN_AU cohort; (c) TCGA_PAAD cohort, Figure S5: Representative protein post-translational modification analyzed in the Figure 4. (a) Total protein carbonylation assessed by in-gel fluorescence quantification (right panel, cy5-DNPH) normalized to the total protein expression assessed by the “stain-free technology” of Biorad^®^ (left panel). (b) HNE post-translational modification of proteins analyzed by western blot (right panel—IB anti-HNE). The average total post-translational modification was normalized on the total protein expression assessed by the “stain-free technology” of Biorad^®^ (left panel). (c) CML post-translational modification of proteins analyzed by western blot (right panel—IB anti-CML). The average total post-translational modifications were normalized on the total protein expression assessed by the “stain-free technology” of Biorad^®^ (left panel). (d) Western Blot analysis of CAT, SOD, and TRDX normalized by β-actin expression. The measured relative intensity is specified beneath each fluorescence acquisition and immunoblot. Table S1: Antibodies used in this study, Table S2: Clinical features of the multi-centric cohort of this study with VEOP < 45 y.o., Table S3: Clinical features of the fused TCGA_PAAD and ICGC_PAN_AU cohort of EOP < 55 y.o., Table S4: Uni- and multivariate analyses of OS and PFS of EOP and LOP from the TCGA_PAAD and ICGC_PAN_AU, Table S5: Mutational profile of *KRAS*, *TP53*, *CDKN2 A,* and *SMAD4* in tumoral tissues from patients in our multicentric cohort, Table S6 Clinical features of patients included in proteomic analysis by 2D-DIGE, Table S7: Identified proteins from 2D-DIGE experiments.
