# Supplementary figures and images for: Pancreatic Ductal Adenocarcinoma Arising in Young and Old Patients Displays Similar Molecular Features
Source: Cancers (Basel). 2021 Mar 11;13(6):1234. doi: 10.3390/cancers13061234 (PMC7999057; doi:10.3390/cancers13061234)

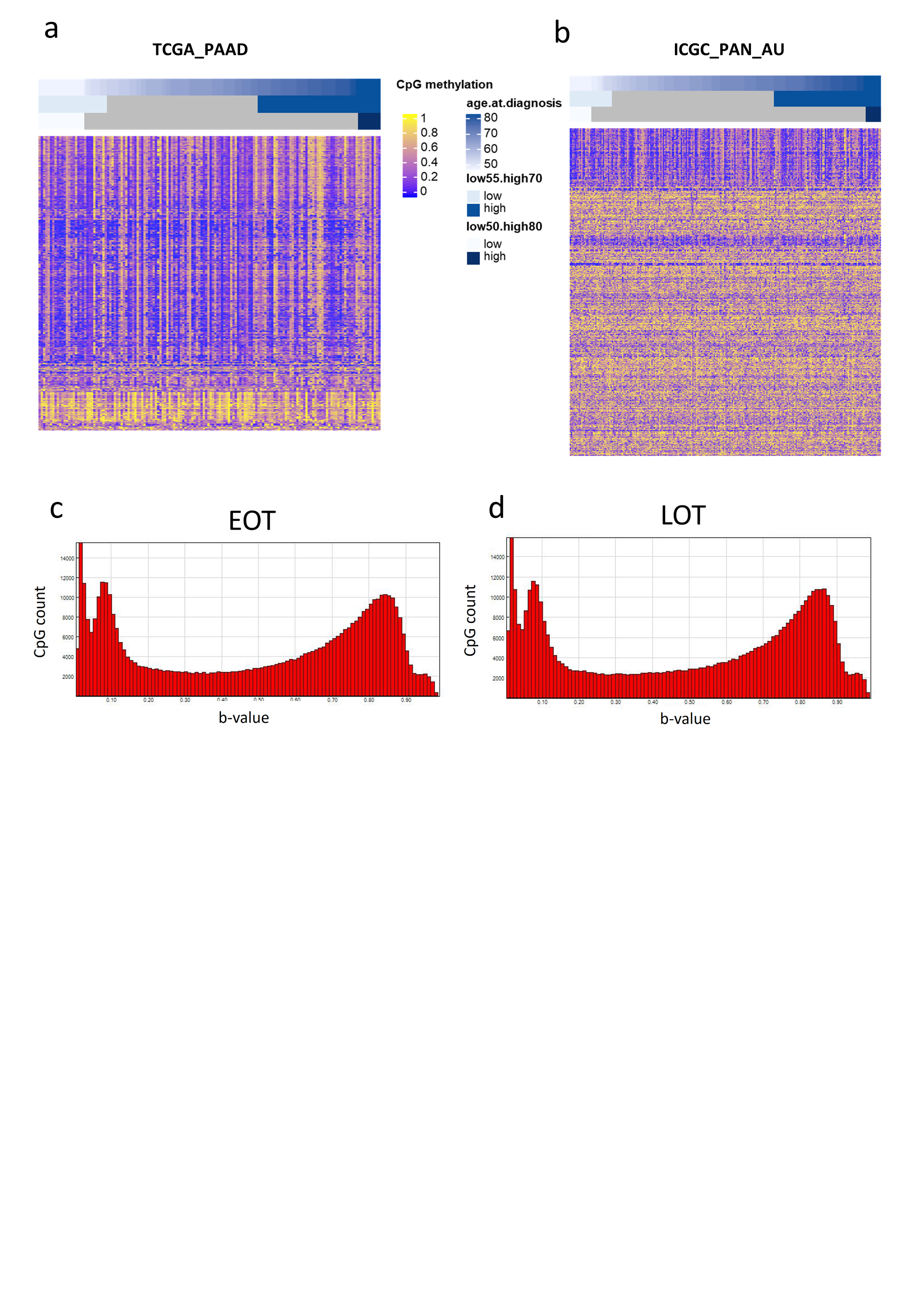

Supplement: Supplementary file 1 [file cancers-13-01234-s001.zip › Figure S/Figure S3.tif]

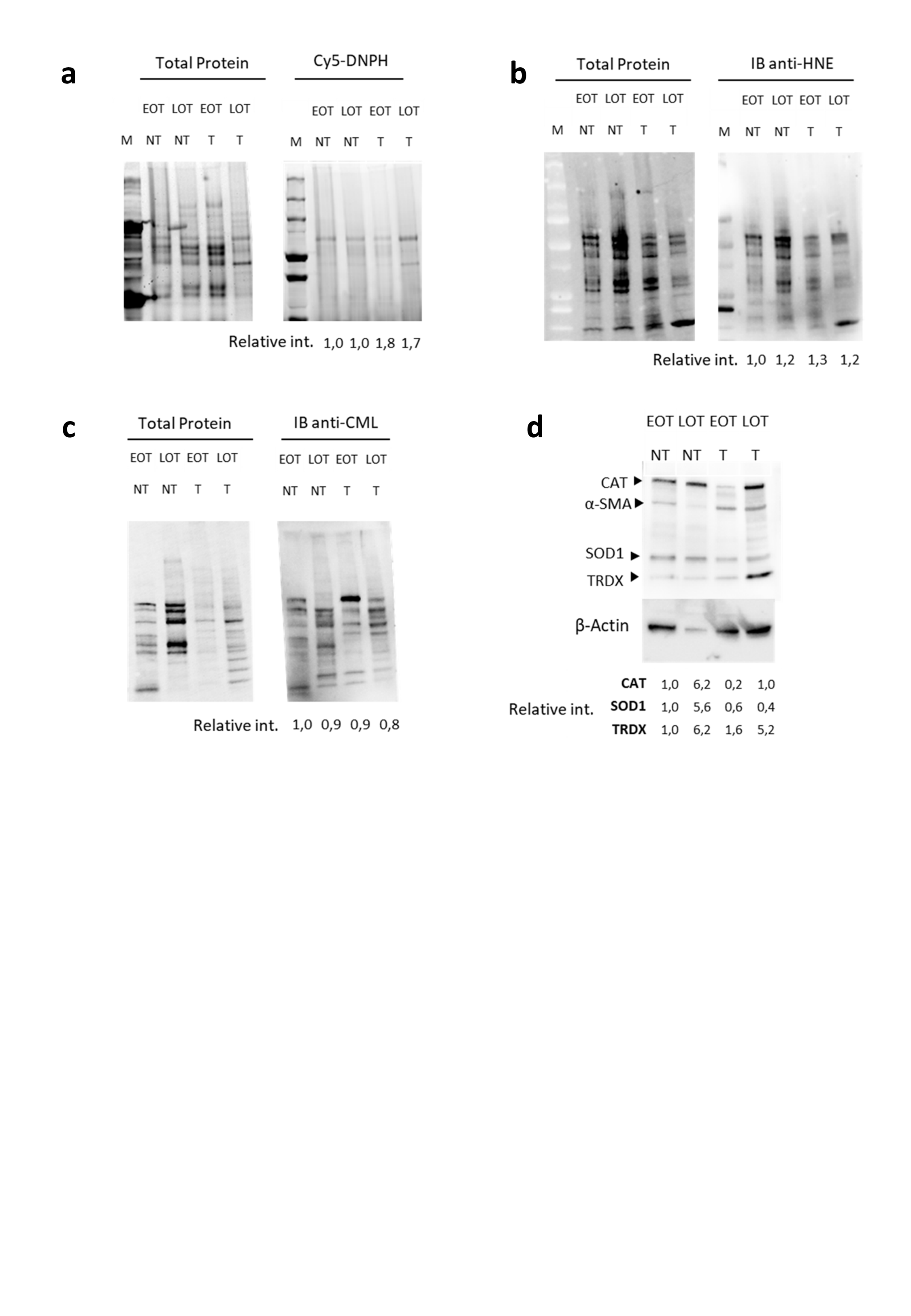

Supplement: Supplementary file 1 [file cancers-13-01234-s001.zip › Figure S/Figure S5.tif]

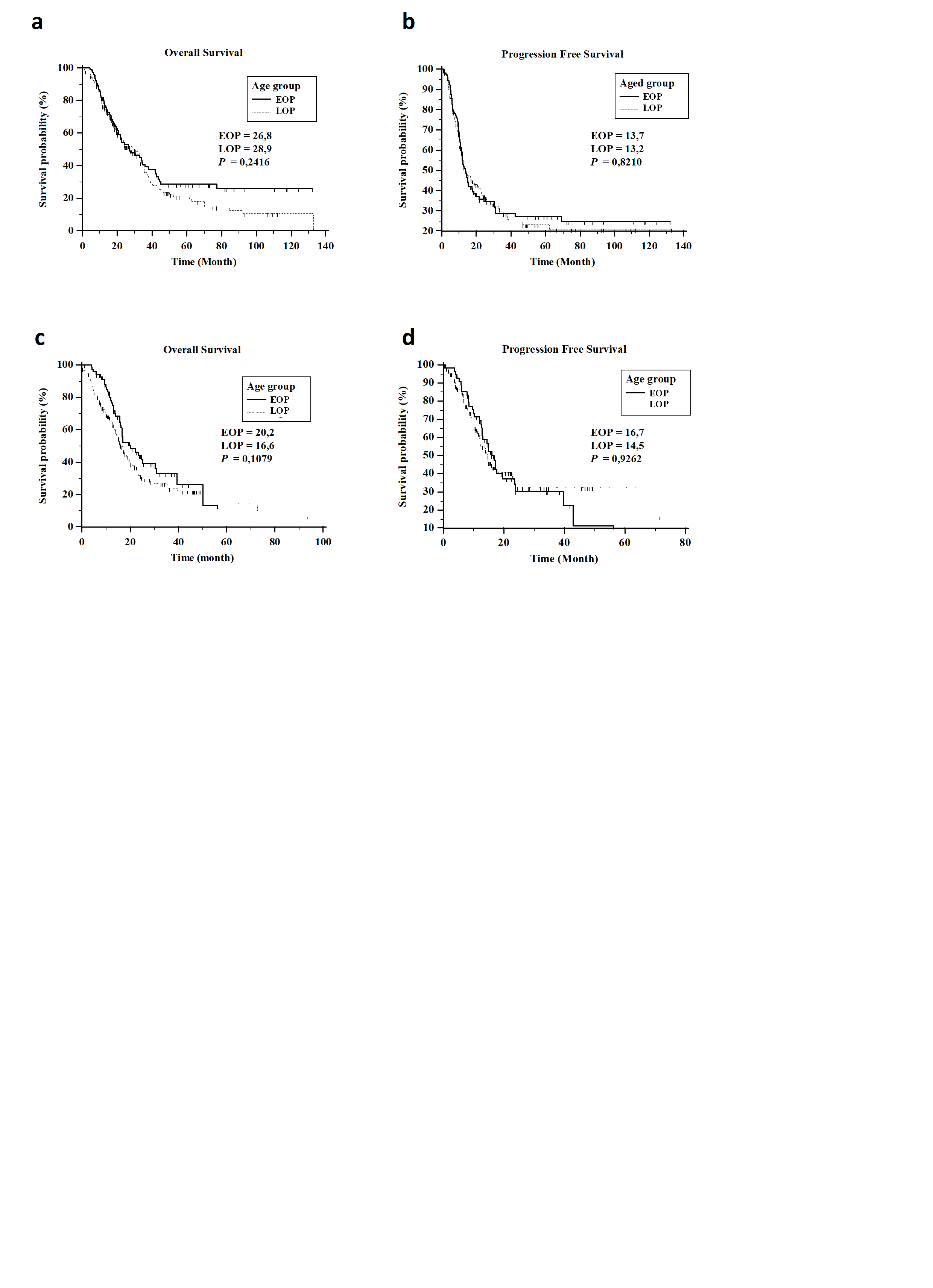

Supplement: Supplementary file 1 [file cancers-13-01234-s001.zip › Figure S/FigureS1.tif]

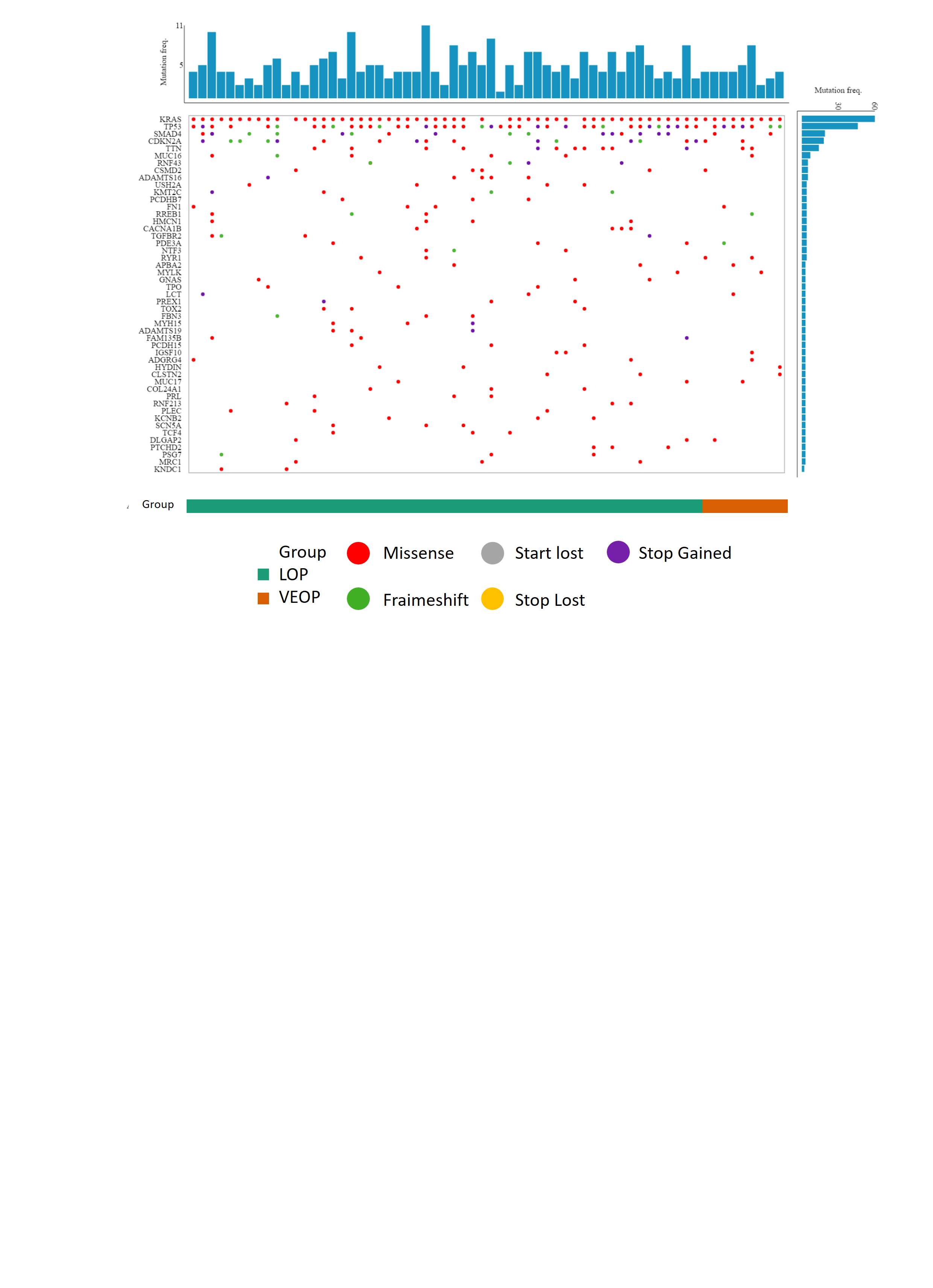

Supplement: Supplementary file 1 [file cancers-13-01234-s001.zip › Figure S/FigureS2.tif]

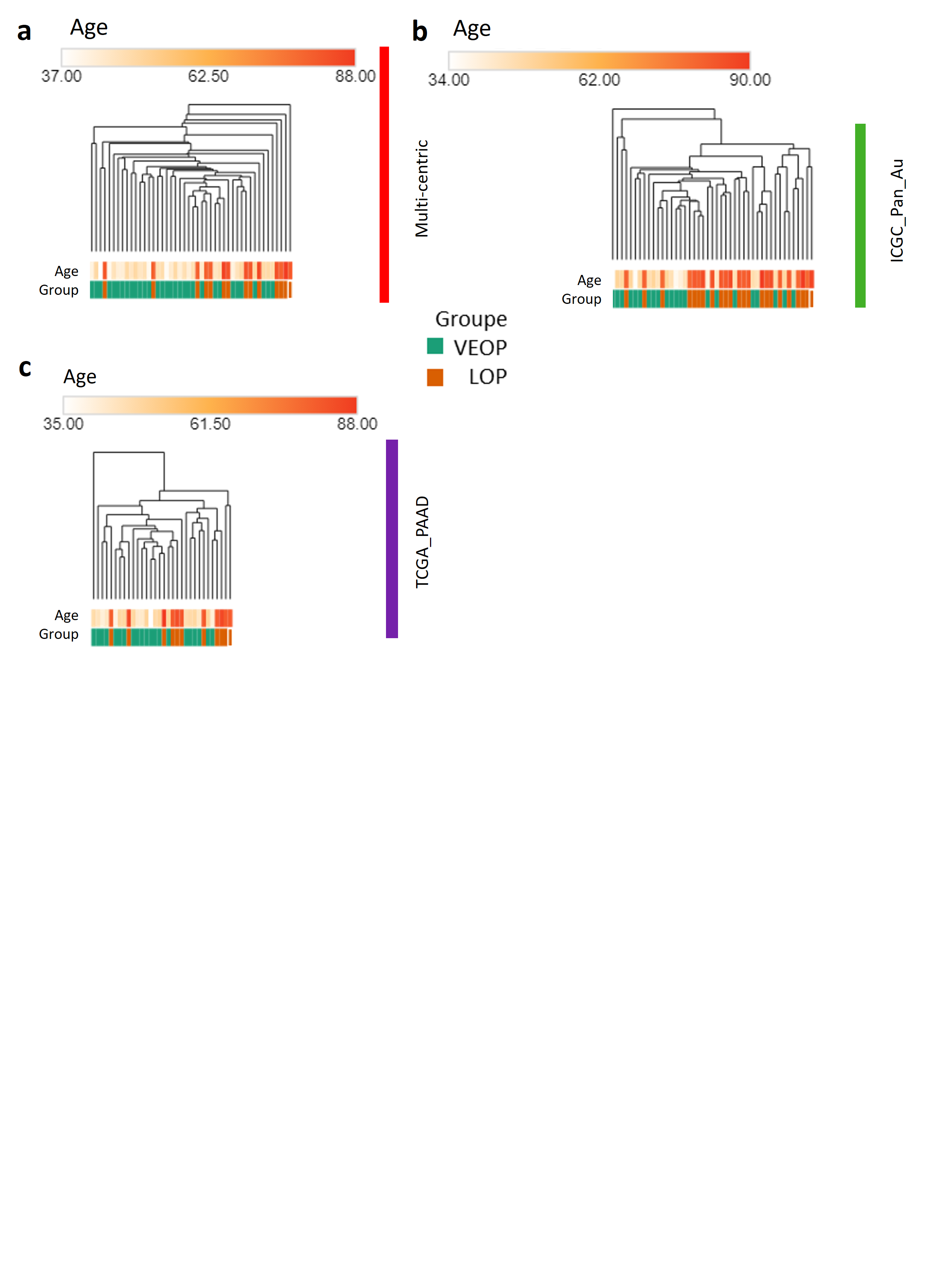

Supplement: Supplementary file 1 [file cancers-13-01234-s001.zip › Figure S/FigureS4.tif]
